# Effects of Aesthetic Chills on a Cardiac Signature of Emotionality

**DOI:** 10.1371/journal.pone.0130117

**Published:** 2015-06-17

**Authors:** Maria Sumpf, Sebastian Jentschke, Stefan Koelsch

**Affiliations:** Cluster “Languages of Emotion”, Freie Universität Berlin, Berlin, Germany; Cognitive Brain Research Unit, FINLAND

## Abstract

**Background:**

Previous studies have shown that a cardiac signature of emotionality (referred to as E_K_, which can be computed from the standard 12 lead electrocardiogram, ECG), predicts inter-individual differences in the tendency to experience and express positive emotion. Here, we investigated whether E_K_ values can be transiently modulated during stimulation with participant-selected music pieces and film scenes that elicit strongly positive emotion.

**Methodology/Principal Findings:**

The phenomenon of aesthetic chills, as indicated by measurable piloerection on the forearm, was used to accurately locate moments of peak emotional responses during stimulation. From 58 healthy participants, continuous E_K_ values, heart rate, and respiratory frequency were recorded during stimulation with film scenes and music pieces, and were related to the aesthetic chills. E_K_ values, as well as heart rate, increased significantly during moments of peak positive emotion accompanied by piloerection.

**Conclusions/Significance:**

These results are the first to provide evidence for an influence of momentary psychological state on a cardiac signature of emotional personality (as reflected in E_K_ values). The possibility to modulate ECG amplitude signatures via stimulation with emotionally significant music pieces and film scenes opens up new perspectives for the use of emotional peak experiences in the therapy of disorders characterized by flattened emotionality, such as depression or schizoid personality disorder.

## Introduction

The massive effects of emotional processes, emotional stress and affective traits on the temporal aspects of cardiac function as reflected in heart rate (HR) and heart rate variability (HRV) have been intensively studied particularly due to their relevance for cardiovascular disease [1–3]. Besides temporal information, the electrocardiogram (ECG) also provides information in terms of cardiac wave morphologies and amplitudes, which is often used in medical practice because the morphology of cardiac P-/T-waves and QRS-complexes is diagnostically relevant in patients with cardiac disease [4, 5]. Importantly, even moment-to-moment amplitude changes have been investigated with regard to their diagnostic relevance. For example, T-wave amplitude variations in the microvolt range (T-wave alternans) were found to be a predictor of sudden cardiac death in patients with ischemic heart disease [6]. Meanwhile it has been found that cardiac amplitudes also carry valuable information about personality traits that play a major role in the processing, experience and expression of emotion [7–9]. The present study investigates transient changes in cardiac amplitudes during intense emotional responses to pleasant stimuli.

Flattened affectivity, an emotional personality trait that is characterized by the reduced ability to experience and express positive, tender feelings, is related to a specific ratio of cardiac amplitudes, referred to as E_κ_, in the resting ECG [7, 8] (see also below). The relation between emotionally relevant personality traits and cardiac amplitudes is thought to be an effect of the modulatory influence of brain structures implicated in emotion (such as orbitofrontal cortex, insular cortex, and amygdala) on the heart, mainly via the autonomic and the endocrine system. Regional heart activity is directly and indirectly modulated by emotional factors via several pathways: (1) Parasympathetic and sympathetic nerve fibers project onto nerve cells within the cardiac nerve plexus [10]. (2) Limbic/paralimbic forebrain structures such as amygdala, insular cortex, and (medial) orbitofrontal cortex modulate the efferent autonomic outflow to the heart [11]. (3) Hormones, like adrenaline and angiotensin II, modulate the activity of neurons in intrathoracic autonomic ganglia [11] (for details see also [7, 8, 12]).

**Fig 1:**
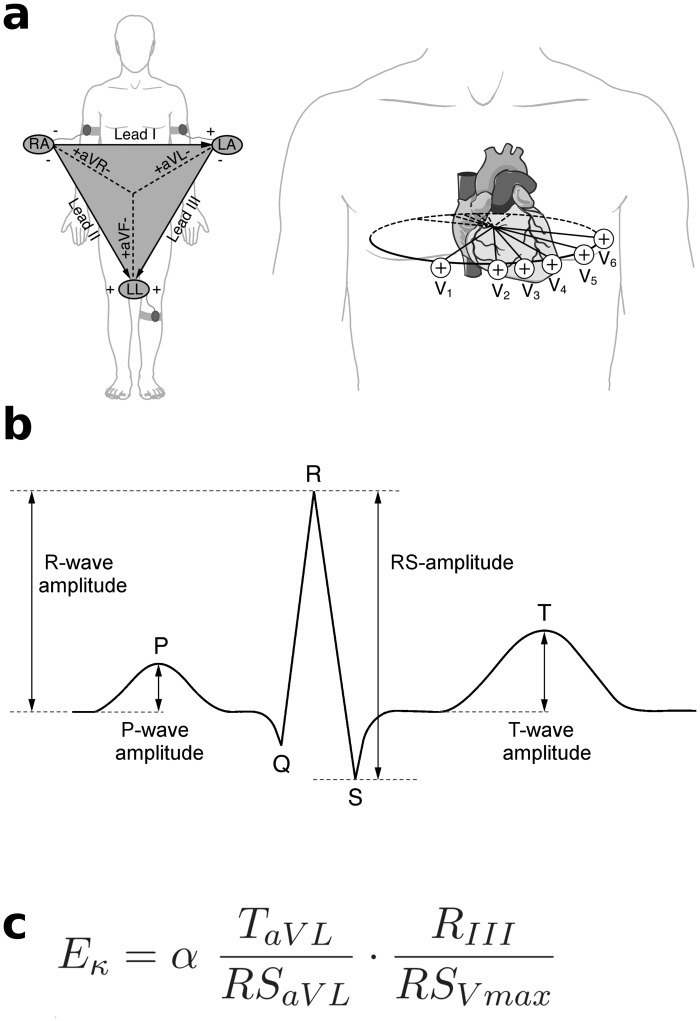
Illustration of the 12 standard ECG leads, ECG waves and E_κ_ values. **(a)** Illustration of standard ECG leads. The six extremity leads (Lead I, Lead II, Lead III, aVR, aVF, aVL) record electrical voltages in different angles on the frontal plane of the body. The six chest leads (V1–V6) record electrical voltages in different angles on a transverse section of the chest. **(b)** Schematic illustration of a heart beat in the healthy ECG. The P-wave reflects atrial depolarization, the QRS-complex ventricle depolarization, and the T-wave ventricle repolarization. **(c)** From four cardiac amplitude values, a single E_κ_ value is calculated. Scaling factor *α* = 10 was introduced for readability reasons.

The ECG amplitude signature E_κ_ was developed in a previous study [7]. Flattened as opposed to normal affectivity (as assessed by the Revised Toronto Alexithymia Questionnaire, TAS-26 [13], and by narrative interviews) could be significantly predicted on the basis of four amplitude values [7]. With the equation shown in Fig 1c, a single value, referred to as E_κ_ (which is explained in detail elsewhere [7]), was computed from these ECG amplitudes for each individual. The E_κ_ value was suitable to distinguish the group quartile with low (flattened) emotionality from the group quartile with high emotionality [7]. Furthermore, E_κ_ values were found to be correlated with autonomic balance: individuals with lower E_κ_ values showed a lower HRV, higher low-frequency (LF) and lower high-frequency (HF) spectral power, as well as a higher LF/HF ratio than individuals with higher E_κ_ values [7, 8]. A similar HRV pattern has been reported previously in studies investigating patients with major depression and pathologic anxiety [14–17]. Using functional magnetic resonance imaging (fMRI) it was observed that E_κ_ values predicted functional differences of brain activity in the amygdala [7] and the hippocampal formation [7, 9] during the processing of emotionally significant musical stimuli: individuals with lower E_κ_ values showed weaker changes of the blood oxygen level dependent (BOLD) signal in response to music stimuli in these structures compared to individuals with higher E_κ_ values [7], and eigenvector centrality of the hippocampal formation was observed to be lower in individuals with lower E_κ_ values [9]. In addition, the latter study [9] showed reduced hippocampal volume in individuals with lower E_κ_ values (compared to individuals with higher E_κ_ values). Finally, E_κ_ values were observed to correlate with an external measure of personality, namely positive emotion [8, 9], a facet of the extraversion scale from the Revised NEO Personality Inventory (NEO-PI-R) [18], which is inversely related to the concept of flattened affectivity. Individuals with lower E_κ_ values had lower scores of positive emotion than individuals with higher E_κ_ values. The reviewed studies indicate that cardiac amplitudes carry information about the emotional personality of an individual. Specifically, low E_κ_ values predict flattened affectivity, and high E_κ_ values predict the tendency to experience and express positive emotion. While the exact mechanisms that lead to inter-individual differences in E_κ_ values remain to be specified, the evidence reviewed above [7, 8] suggests that low E_κ_ values primarily reflect autonomic imbalance as a correlate of flattened affectivity.

The present study investigates whether the cardiac signature E_κ_ can be modulated by evoking intense pleasure, as manifested in emotional piloerection during so-called “aesthetic chills” (also referred to as “thrills” or “frissons”). As stimuli we used pleasant music pieces and emotionally touching film scenes. The phenomenon of aesthetic chills is a well-established marker of peak emotional responses [19–30]. The first study to investigate chills [30] (terming them “thrills”), found them described unanimously by 585 questionnaire respondents as a “shudder, tingling, or tickling. It may be accompanied by a feeling of ‘hair standing on end’ or ‘goose bumbs’ on the arms” [30] (p. 127). It was reported to be related to very intense feelings, most frequently during film scenes and musical passages [30]. The phenomenon has since then repeatedly been used to precisely index moments of peak pleasure when studying music-evoked emotion [21, 26, 28, 29, 31, 32], and has consistently been found to correlate with physiological markers of increased arousal. A transient increase in HR [21, 28, 31, 32], as well as increased skin conductance level (SCL) [23, 28, 31, 33] during chills were found consistently across several studies.

Because chills are due to activity of the sympathetic nervous system [34], one would expect them to also correlate with increases of the respiratory frequency (RF) [34]. Reports of RF changes during chills are, however, not consistent in the literature [28, 31, 32]. Finally, chills are also related to affective personality traits: In a study by Rickard [22], the mean number of experienced chills per minute was positively correlated with the NEO-FFI extraversion scale.

In most previous studies, the measurement of chills has relied on subjects’ reports [19, 21–23]. This has the disadvantages of (1) posing a self-monitoring task on subjects, which can distract them from the stimulus and interfere with the experience of intense emotions, and (2) being prone to self-perception errors [32, 35]. For these reasons, and based on the fact that chills are in many (though not all) cases accompanied by piloerection (or “goosebumbs”), a piloerection camera and analysis software (the “Goosecam”) was developed and introduced by Benedek and colleagues [35], which was used in this study in addition to gathering the subjective reports. The Goosecam produces video recordings (e.g. of the forearm skin) which are then analysed by means of spectral analysis (2D discrete Fourier transformation), thereby objectively detecting chills accompanied by piloerection [32, 35]. Because chills with piloerection and chills without piloerection might comprise two distinct psychological phenomena, we were interested in the peripheral-physiological correlates of both. Based on the reported previous findings [7–9] we hypothesized that E_κ_ values, which are related to positive emotion, are transiently modulated by aesthetic chills (i.e., by strongly positive emotion) as indicated by piloerection. We therefore expected a relative increase of E_κ_ values at, or shortly after, the onset of aesthetic chills. We also measured HR and RF to render our data comparable with literature descriptions of the physiological effects of chills, with the hypothesis of a relative increase in HR during the chill passage [21, 28, 31, 32]. No directed hypotheses were made regarding RF changes during chills, and regarding the difference in peripheral-physiological correlates between chills with and without piloerection. Based on Rickard (2004) [22], we further hypothesized that individuals who frequently experience chills (i.e., intense positive emotions) have higher scores of extraversion than the normal population.

## Methods

### Subjects

Participants were recruited with an advertisement specifically directed towards individuals who often experience chills while listening to music or watching film scenes [31], because in many individuals the chill phenomenon is a rather rare event, and to some it is even unknown [19, 20, 30]). Measurements from 58 subjects (34 females), aged between 19 and 38 years (*M* = 28.34, *SD* = 5.24), 67% University students, were collected. According to self-report, all of them had normal hearing and no diagnosed cardiovascular disease, nor any mental or psychiatric disorder. The study was conducted in accordance with the guidelines of the Declaration of Helsinki, approved by the ethics board of the Department of Psychology and Educational Sciences of the Freie Universität Berlin, and written informed consent was obtained from all participants.

### Stimulus selection and matching of stimulus pairs

In order to maximize the probability of chill events, subjects were asked to bring their own stimulus material [19, 22, 27, 29–31, 36]. In pilot studies we found a heterogeneous preference of our subjects for either music or film stimuli, suggesting that in many cases, one but not the other would be effective in evoking chills. Hence, again in order to maximize the probability of chills, both stimulus types (music and film) were used. Each subject brought two music pieces (played in full-length [27]) and one film scene (presented with a length of approximately 10 minutes), with no restrictions on genre [19, 22, 27, 31]. Each subject’s selected chill stimulus was used as another subject’s “neutral” control stimulus (henceforth referred to as “matched pair”) [21, 29, 31, 32]. This results in identical chill and control stimulus sets for comparative group analyses and thus excludes the possibility that found effects are solely due to visual/acoustic feature differences of the stimuli.

### Procedures

Participants were tested individually. The session began with filling out the German version of the NEO-FFI questionnaire [18]. During the subsequent stimulus presentation and physiological measurement phase they lay comfortably in supine position, wearing headphones and video goggles (Zeiss Cinemizer Plus, Carl Zeiss, Oberkochen, Germany). To avoid piloerection induced by cold temperature, the temperature in the experimental room was set to a comfortable level for the participant.

The stimulus presentation phase consisted of two self-selected music pieces, two control music pieces, one self-selected film scene and one control film scene. The order of stimuli was counterbalanced across participants. Each stimulus was preceded by a 30-seconds resting phase, in which participants were presented with a black screen and instructed to relax, followed by a 12 questions rating phase (see Ratings section). The duration of an experimental session was about 1.5 hours (including about 50 minutes physiological measurement time).

### Chill measurement and analysis

Two conditions of chills were measured: (1) Chills with piloerection (“objective”), and (2) chills without piloerection (“subjective”).

Piloerection was measured during the entire experimental session objectively using the “Goosecam” [35]. The data curves that were extracted from the Goosecam video were manually cleaned from movement artifacts, detrended and filtered (high-pass Butterworth filter, cut-off frequency 0.8 Hz). Piloerection was then detected automatically and offline with the software package “Gooselab” (http://www.goosecam.de/) according to the instructions given in [35].

In addition to this *objective* (Goosecam) measurement, the *subjective reports* of chills experienced during the session were documented. In order to not distract or disturb subjects during the stimulation phase, they were asked only after each stimulus (during the rating phase) about the number and the time of chill incidences. In the course of data evaluation, the reported timing, typically referring to a specific moment in the film scene or a passage in the song, was carefully translated into exact time points. In all cases of insufficient timing information due to imprecise paraphrasing, reported chill events were excluded from further data analysis.

### ECG and ECG-derived measurements and analysis

#### Measurement and preprocessing

The assessed ECG and ECG-derived measures were (1) E_κ_ values, (2) heart rate (HR), and (3) respiratory frequency (RF). We did not calculate heart rate variability (HRV) data, because the recommended minimum length for the calculation of HRV data is usually in the range of minutes [37], whereas the length of a chill is in the range of seconds [24]. A standard 12 leads ECG was recorded in supine position during the entire measurement. Measurement conditions were in accordance with the guidelines of the Task Force of the European Society of Cardiology and the North American Society of Pacing and Electrophysiology [37]. ECGs were recorded with a sampling rate of 1000 Hz. ECG raw data were analysed with the in-house software package Kardionoon 2.0 [7], which performs detection of ECG-waves and measurement of absolute P-, R-, RS-, and T-wave amplitudes, as described elsewhere [7, 8]. An illustration of standard ECG leads and of P-, R-, RS-, and T-wave amplitudes is provided in Fig 1a and 1b. E_κ_ values were then computed continuously for each individual across the whole course of measurement time, according to the equation in Fig 1c. HR and RF were computed from the ECG signal. HR was defined as 60/NN bpm (with NN being the interval of successive normal heart beats in seconds) and was calculated from the detected R-peaks. ECG-based RF curves were obtained using the Matlab-based ECG-derived respiration (EDR) algorithm [38] based on estimated, respiration-evoked rotation angles of the heart’s electrical axis (for a detailed description of the algorithm, see [38]). Estimating the RF from the ECG has the advantage of obviating the need for any additional and likely uncomfortable measurement devices, like abdomen- and chest-belts.

Movement artifacts were eliminated manually, and all data curves were downsampled to 1 Hz to obtain identical sampling for all data curves (E_κ_, HR, RF) [32]. All physiological data were normalized (*z*-standardization) within participants before entering the analysis.

#### Physiological short-term responses around the onset of chills

Chill/piloerection passages with 15-seconds pre- and post- chill/piloerection onset (i.e., 31 1-Hz-samples) were extracted from the data. Control passages based on identical stimulus material were taken from the “matched partner” subjects, who did not have chills/piloerection during the entire stimulus.

Baseline activity was defined as the activity at -15 to -5 s (i.e., the first ten seconds of the 31-seconds passages), because physiological activity already starts to change about 5 seconds before piloerection onset [32]. Each sampling point (of one second length) of the chill/piloerection phases was compared to baseline activity by means of a series of 31 T-tests. This was done separately for E_κ_, RF and HR, and separately for chills with and without piloerection, resulting in six comparisons in total. To account for multiple comparisons in the T-test series, ordered Bonferroni correction was applied to the alpha value. This alpha value correction method is specifically suited for multiple comparisons which are not independent from each other [39].

#### Average physiological change

The average physiological activity (E_κ_, RF and HR) during each entire piloerection passage (which differed in length) was also compared to a random no-piloerection control passage of the same length and within the same stimulus and subject. Those passage pairs differ not only in terms of the elicited physiological activity, but also in terms of visual/acoustical characteristics. Therefore, the average relative change (piloerection passage minus random control passage from the same stimulus, henceforth “difference score”) was compared to the two identical periods in a matched partner subject. This enabled us to assess the physiological changes while controlling for stimulus properties (which were the same in the subject with piloerection and the matched control). If and only if piloerection was accompanied by physiological changes and these changes were due to piloerection alone (and not due to visual/acoustic stimulus features), then the magnitude of the difference score of the participant with piloerection would be larger than the one of the matched partner. The comparison of (1) E_κ_, (2) RF and (3) HR piloerection / matched control scores was done by means of three independent T-tests.

### Rating

After each stimulus, participants were asked to tell the number and point in time of chills they had experienced during the stimulus. Then they were asked to rate the stimulus with respect to the experienced emotional intensity, valence and emotional quality on 7-point Likert scales, to ensure that the chill-evoking stimuli were in fact perceived as pleasurable and connected to positive, not negative, emotion [19, 40]. To assess emotional quality, 9 feeling terms were selected from the Geneva Emotional Music Scale (GEMS) [41] (one term out of each of the 9 GEMS musical emotion factors: wonder, transcendence, tenderness, nostalgia, peacefulness, power, joyful activation, tension, and sadness), translated into German. To identify chills which might have been evoked by sad, stirring film scenes, which would undermine positive emotion, the item “perturbed” (“verstört”) was added. Thus, there were 12 rating items in total (listed in Table 1). They were compared between chill-evoking stimuli and not-chill-evoking stimuli by means of Mann-Whitney *U* tests, on a Bonferroni-corrected significance level 0.05/12 = 0.0042.

**Table 1:** Poststimulus ratings (English translation). Asterisks indicate significant differences between stimuli evoking chills and stimuli not evoking chills (corrected for multiple comparisons).

Topic	Item (all Likert 1–7)	chill M / SD	no chill M / SD	Mann-Whitney U U / p
Emotional Intensity	“How intense was your emotion while you listened to the piece / watched the scene?”	5.4 / 1.4	3.1 / 1.6	3496.5 / 0.000*
Emotional Valence	“How pleasant was it for you to listen to the piece / watch the scene?”	5.5 / 1.6	3.9 / 1.8	5491.0 / 0.000*
Emotional Quality	“How did you feel during the most intense phases of the piece / the scene?”			
	“happy” (GEMS factor wonder)	4.3 / 1.8	2.8 / 1.6	5952.5 / 0.000*
	“inspired” (GEMS factor transcendence)	4.2 / 1.8	2.6 / 1.5	5744.0 / 0.000*
	“tender” (GEMS factor tenderness)	4.5 / 1.7	2.9 / 1.7	5731.0 / 0.000*
	“melancholic” (GEMS factor nostalgia)	4.2 / 1.9	2.8 / 1.7	6717.5 / 0.000*
	“calm” (GEMS factor peacefulness)	3.8 / 1.6	4.2 / 1.7	9481.0 / 0.031
	“energetic” (GEMS factor power)	3.8 / 1.8	2.4 / 1.5	6076.5 / 0.000*
	“amused” (GEMS factor joyful activation)	3.2 / 1.9	2.5 / 1.6	8873.0 / 0.003*
	“tense” (GEMS factor tension)	3.5 / 1.8	2.7 / 1.8	7929.5 / 0.000*
	“sad” (GEMS factor sadness)	3.3 / 2.0	2.3 / 1.7	7935.0 / 0.000*
	“perturbed”	1.8 / 1.3	2.0 / 1.6	10715.5 / 0.523

### Personality questionnaire

Personality scores were assessed with the German translation of the NEO Five Factor Inventory (NEO-FFI) [18]. Standard scores of the personality traits neuroticism, extraversion, openness to experience, conscientiousness, and agreeableness in our study population were compared to a) the norming sample (with standard score *SS* = 100) and b) an independent group of 159 healthy subjects who were matched with our experimental group with respect to age and gender distribution, height, weight, BMI, as well as educational level. This was done because our sample had a bias towards University education, whereas the German norming sample of the NEO-FFI is balanced with respect to educational level [42]. Personality standard scores were further compared between subjects showing piloerection and subjects reporting chills, but not showing piloerection. All comparisons were done by means of independent T-tests.

## Results and Discussion

### Chills and piloerection incidences

All 58 subjects reported subjective experiences of chills. However, only 25 of our 58 subjects (43.1%) had measurable piloerection during the course of the measurement (this ratio is consistent with previous findings [32]). In total, 966 subjectively experienced chills were reported, and 568 piloerection incidences were measured. Hence, subjects reported roughly 1.7 times as many chills as they had measurable piloerection incidences, which is consonant with other studies [27, 32]. Of the 966 reported chills, 492 could be accurately located in time. Music and film stimuli were equally effective with respect to piloerection incidences (52.7% of which occurred in response to music), but not with respect to subjectively perceived chills (82.7% of which occurred in response to music).

Piloerection incidences and chills often occurred in series, rapidly following each other with few seconds distance. Of all those series we used only the first incidence, to rule out overlap and interference of effects. 139 chills and 93 piloerection incidents remained. The number of measured piloerection incidences and the number of reported chills in each stimulus were correlated (*R* = 0.24, *p* < .001).

83 (of the 139) chills and 69 (of the 93) piloerection incidences could successfully be matched to the corresponding stimulus passage in the matched partner subject, i.e., the matched partner subject did not have chills/piloerection in that piece or scene. These were used in the further analysis.

### Physiological short-term responses around the onset of chills

**Fig 2:**
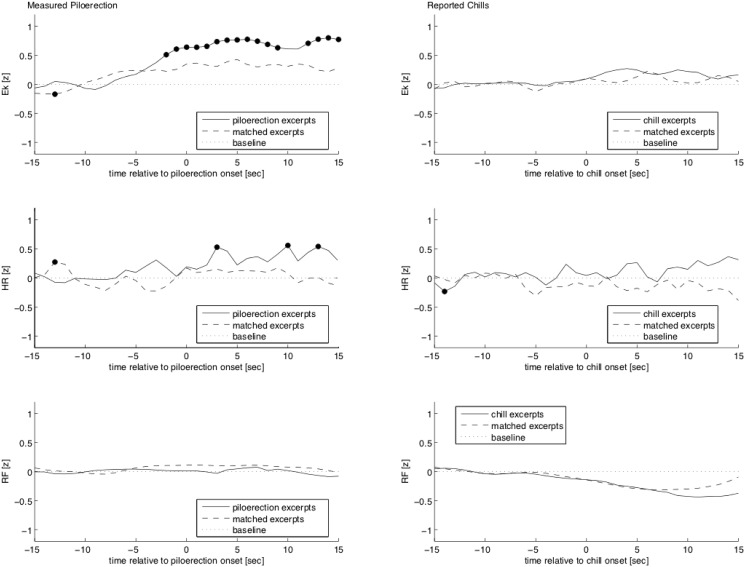
Time course of the chill response with piloerection (left column) and without piloerection (right column), 15 seconds before and after chill/piloerection onset (= 0*s*). The first ten seconds of each period (-15 to -5 s period before piloerection onset) were considered as the baseline, and their mean value was subtracted from the entire passages (resulting in a zero baseline, depicted by the dotted line). The solid line depicts the response of subjects who experienced piloerection/chills. The dashed line depicts the data of subjects who did not experience piloerection/chills in the same stimulus passages. Black dots mark significant deviations from zero/baseline. Ek = emotional index E_κ_, HR = heart rate, RF = respiratory frequency.

Music and film stimuli were pooled in the following analyses, because effects of separate stimulus types (music or film) were, albeit descriptively similar to the effects found when pooling them, mostly not significantly different from zero. Fig 2 shows the physiological responses to chills with piloerection (left column) and chills without piloerection (right column), compared to the physiological activity of the matched partner subjects (who did not have chills nor piloerection during the same musical or film passage). Fig 2a shows the time course of normalized E_κ_ values, Fig 2b shows the normalized heart rate, and Fig 2c shows the normalized ECG-derived respiratory frequency.

#### *E_K_* values

During chills with piloerection, E_κ_ values increased significantly between -2 and 8 seconds relative to piloerection onset (*t* >= 3.37, *p* <= 0.006 for each test), and between 12 and 14 seconds after piloerection onset (*t* >= 3.38, *p* <= 0.004 for each test). This shows that E_κ_ values, and thus regional cardiac activity (as reflected in the cardiac amplitude signature), change during the course of chills with piloerection. In the matched passages (dashed line of Fig 2a), E_κ_ values increased slightly, but not significantly. Given the facts that, firstly, matched partner subjects were presented with identical stimuli, and secondly, control stimuli were not required to be rated as neutral or negative by the subjects, it is reasonable to assume that, to a lesser extent, subjects were emotionally and physically responding also to control pieces and scenes. No significant changes in E_κ_ were found during chills without piloerection (right column of Fig 2). Thus, E_κ_ values increased significantly during chills with piloerection, but not during chills without piloerection.

#### Heart rate (HR)

The HR increased significantly after piloerection onset (Fig 2, solid line). This increase was significant at 3 (*t* = 3.71, *p* < 0.001), 10 (*t* = 3.53, *p* < 0.001) and 13 (*t* = 3.33, *p* = 0.002) seconds after piloerection onset. The finding of a HR increase following piloerection onset is consistent with previous studies reporting a HR increase during music-evoked chills [21, 28, 29, 31, 32]. In the matched passages (Fig 2, dashed line), HR exceeded the average baseline value once at -13 seconds (*t* = 2.76, *p* = 0.008). There was no significant increase in HR during chills without piloerection. Thus, as the E_κ_ values, HR increased during chills with piloerection, but not during chills without piloerection. It is unlikely that the increase of E_κ_ was merely a consequence of the accelerated HR, because significance of the increase was reached earlier for E_κ_ values than for HR values. This assumption is consistent with a previous study reporting that E_κ_ values and HR are unrelated in the rest ECG [7].

#### Respiratory frequency (RF)

There were no significant effects of chills on RF. Earlier studies have found a) no changes in RF during piloerection [32], b) a very brief RF increase at 1.1 seconds before chill onset [28], and c) substantially increased RF directly at chill onset, sharply decreasing again 3 seconds later [31]. Reasons for these inconsistencies remain to be clarified.

Taken together, E_κ_ was modulated by peak positive emotions as indicated by chills with piloerection during the exposure to music pieces and film scenes. Chills with piloerection were also accompanied by an increase in HR (which is in line with previous literature [21, 28, 29, 31, 32]). Thus, our results suggest that the increase in E_κ_ is associated with an increase in sympathetic activity, as indicated by increased HR. Physiological changes (E_κ_, HR) were significant (at more than one point in time) in the presence of piloerection, but not during the subjectively reported chills without piloerection. This suggests that the phenomenon of piloerection is a physiological intensification of the mere chill experience. In other words, a chill experience may in the extreme (and more rare) case exceed an intensity threshold and lead to strong physiological responses (piloerection, HR acceleration, E_κ_ value increase), while in the less extreme (and more frequent) case it remains below that threshold. To clarify this conclusively, a study design that allows for a more exact localization of subjective chill experiences in time would be needed.

### Average physiological change

**Fig 3:**
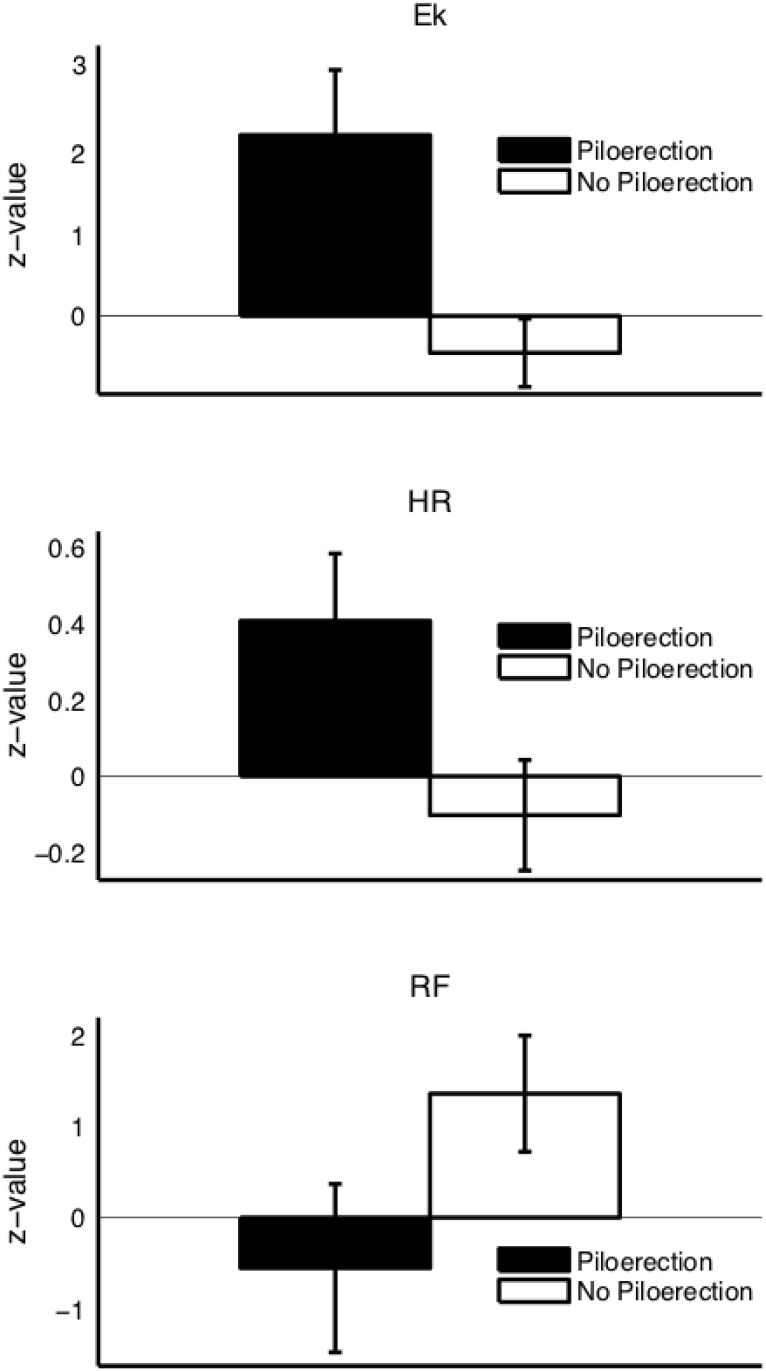
Average physiological changes in participants who had piloerection (black bars) and their matched partners (white bars). Black bars depict the difference in physiological activity between the entire piloerection passage and a random control passage of similar length within the same stimulus and subject. White bars depict the same difference scores, but for matched partner subjects who did not have piloerection in either of the passages. All scores are normalized with respect to activity during the baseline period. Ek = emotional index E_κ_, HR = heart rate, RF = respiratory frequency. HR and E_κ_ scores are different from zero in the piloerection condition (black bar), RF scores are different from zero in the no-piloerection condition (white bar). The differences in HR scores and in E_κ_ scores (black versus white bars) are significant on a 0.05 significance level.

In Fig 3, black bars show the “difference score” (see Methods) of participants who had piloerection, i.e., their difference in physiological activity between the entire piloerection passage and a random control passage within the same stimulus. White bars show the “difference score” of matched partners.

#### *E_K_* values

The E_κ_ difference scores were significantly different from zero in participants who had piloerection (*t* = 2.53, *p* = 0.007), but not in their matched partners. The scores of participants who had piloerection and their matched partners furthermore differed significantly from each other (*t* = 2.76, *p* = 0.004). This shows that there was a significant difference in E_κ_ between piloerection passages and random control passages, which was independent of visual/acoustic stimulus feature differences.

#### Heart rate (HR)

The HR difference scores were significantly different from zero in participants who had piloerection (*t* = 2.24, *p* = 0.017, but not in their matched partners. The scores of participants who had piloerection and their matched partners furthermore differed significantly from each other (*t* = 2.24, *p* = 0.03). This shows that there was a significant change in HR between piloerection passages and random control passages, which was independent of visual/acoustic stimulus feature differences.

#### Respiratory frequency (RF)

The RF difference scores were not different from zero in participants who had piloerection. The RF difference scores were different from zero in the matched partners (*t* = 2.12, *p* = 0.04), meaning that there was an unexpected significant difference in RF between two visually/acoustically different passages, both in the absence of chills. There was no significant difference between the difference scores of participants who had piloerection and their matched partners. Thus, the effect of acoustical/visual differences on RF was not further substantiated, and no effect of piloerection on the RF was found.

### Ratings

The values of the 12 emotion ratings are provided in Table 1, together with the test statistics. Pieces and scenes that evoked chills were rated as significantly (1) more emotionally intense, and (2) more pleasant (i.e., higher in valence). During chill-evoking stimuli, subjects felt significantly more “happy”, “inspired”, “tender”, “energetic”, “amused”, “tense”, and also more “sad” and “melancholic”. The items “sad” and “melancholic” (from the GEMS factors “sadness” and “nostalgia”) were significantly correlated with positive valence (Spearman’s *ρ* = 0.18 and Spearman’s *ρ* = 0.43, respectively; *p* <= 0.005), showing that in response to chill-evoking music, positive and negative emotion can be felt simultaneously. This supports the hypothesis that piloerection can be an indicator for an ambivalent emotional state of being moved (either to tears or by awe) [32], which is a state of mixed valence [24, 32, 43] that causes an improvement of mood [24], has a strong social component [24, 32, 44], and may be accompanied by physiological indicators of sadness [32, 45]. Two emotional quality terms were *not* rated higher in the presence of chills: The first one was, as expected, the “perturbed”-item, the intention of which had been to identify upsetting film scenes (moving, but in a negative way). Thus, all chills in our experiment were, as intended, caused by positive emotion. The second term not rated higher in the presence of chills was the item “calm” from the GEMS factor “peacefulness”. This is consistent with results of an earlier study that relates the chills phenomenon to emotional arousal [31].

There were no differences in emotion ratings between chills with piloerection and chills without piloerection.

### Personality questionnaire

With respect to NEO scores, our experimental group was compared to (1) the norming sample, and (2) an independent group of 159 healthy individuals with University students being overrepresented (like in our sample, see Methods). The NEO scores are provided in Table 2. Compared to the norming sample, the subjects from our experiment, i.e. individuals frequently experiencing chills, had higher scores of extraversion (*t* = 3.61, *p* = 0.001), openness to experience (*t* = 11.49, *p* < 0.001), agreeableness (*t* = 3.91, *p* < 0.001) and neuroticism (*t* = 2.54, *p* = 0.014). The relationship between the frequent experience of aesthetic chills and openness has also been found in two previous studies [46, 47].

**Table 2:** NEO-FFI Personality Scores (Std Scores).

	All chill subjects M / SD	Chill subjects with piloerection M / SD	Chill subjects without piloerection M / SD	Independent group M / SD
**Neuroticism**	105.1 / 14.5	102.1 / 16.6	107.5 / 12.3	104.00 / 16.3
**Extraversion**	111.0 / 22.2	117.1 / 20.0	106.0 / 23.15	98.9 / 20.2
**Openness to Experience**	121.0 / 13.3	123.1 / 14.3	119.3 / 12.5	117.6 / 17.8
**Agreeableness**	110.0 / 18.6	117.5 / 19.6	103.8 / 15.5	106.4 / 17.7
**Conscientiousness**	98.6 / 19.2	100.4 / 19.2	97.2 / 19.3	95.5 / 17.4

Compared with the independent, matched group, our experimental group had higher scores of extraversion (*t* = 0.99, *p* < 0.001). Extraversion scores also correlated with the absolute number of chills experienced by each individual in our experiment (Spearman’s *ρ* = 0.28, *p* = 0.046), whereas the correlation between the extraversion scores and the number of chills experienced per minute just failed to reach significance (Sperman’s *ρ* = 0.26, *p* = 0.057). Thus, our data confirm findings of the chills phenomenon being related to extraversion [22] at least in part (in the study by Rickard [22], extraversion correlated with the number of chills per minute). This was an expected result, because chills are markers of strong positive emotion, and the tendency to experience and express positive emotion is reflected in high extraversion scores. Thus, the tendency to experience chills should be reflected in high extraversion scores.

Finally, individuals with piloerection had higher agreeableness scores than individuals who experienced chills without piloerection (*t* = 0.97, *p* = 0.008; see also Table 2).

### Future perspectives

The current experiment demonstrates that E_κ_ values can be transiently modulated by emotional chills that are accompanied by measurable piloerection. This opens a perspective to use cardiac signatures like E_κ_ (and possibly cardiac amplitudes in general) in emotional learning therapy. For example, biofeedback therapies used to treat disorders characterized by flattened emotionality, such as depression or schizoid personality disorder, could be based on the E_κ_ values. Biofeedback therapies for major depression are so far primarily based on electroencephalography (EEG) [48–52]. ECG-derived measures of emotion may provide additional information, while having fewer technical requirements (which makes them easier to apply).

## Conclusion

The cardiac signature E_κ_ reflects inter-individual differences in the tendency to experience and express positive emotion which are relatively stable over time [8, 9]. Our results show that regional cardiac activity, as reflected in the E_κ_ values, can be transiently modulated by emotional chills with piloerection. The chills evoked in our study reflect intensely pleasurable emotional responses to music and film stimuli (associated with increased arousal, as indicated by subjective ratings as well as an increase in heart rate). Hence, our results show that positive emotional chills with piloerection modulate regional cardiac activity. The chill-dependent increase of E_κ_ values was independent of a) visual/acoustical variations in the stimuli, as well as b) other physiological measures (heart rate). The possibility to modulate cardiac amplitudes by evoking peak emotional responses opens perspectives for applications in emotional learning therapy and for further research.

## Supporting Information

S1 Datatable Post stimulus ratings, NEO-FFI scores and ECG data of participants. (SAV)
